# Suitability of Polyacrylamide-Based Dosimetric Gel for Proton and Carbon Ion Beam Geometric Characterization

**DOI:** 10.3390/gels11100794

**Published:** 2025-10-02

**Authors:** Riccardo Brambilla, Luca Trombetta, Gabriele Magugliani, Stefania Russo, Alessia Bazani, Eleonora Rossi, Eros Mossini, Elena Macerata, Francesco Galluccio, Mario Mariani, Mario Ciocca

**Affiliations:** 1 Department of Energy, Nuclear Engineering Division, Politecnico di Milano, 20156 Milano, Italy; 2 Department of Medical Physics, Fondazione CNAO (Centro Nazionale di Adroterapia Oncologica), 27100 Pavia, Italy

**Keywords:** polymer gel dosimetry, hadron therapy, quenching, MRI, dose profiles, geometric beam characterization

## Abstract

Experimental measurement of dose distributions is a pivotal step in the quality assurance of radiotherapy treatments, especially for those relying on high delivery accuracy such as hadron therapy. This study investigated the response of a polymer gel dosimeter to determine its suitability in performing geometric beam characterizations for hadron therapy under high-quenching conditions. Different extraction energies of proton and carbon ion beams were considered. Gel dose–response linearity and long-term stability were confirmed through optical measurements. Gel phantoms were irradiated with pencil beams and analyzed via magnetic resonance imaging. A multi-echo T_2_-weighted sequence was used to reconstruct depth–dose profiles and transversal distributions acquired by the gels, which were benchmarked against reference data. As expected, a response-quenching effect in the Bragg peak region was noted. Nonetheless, the studied gel formulation proved reliable in acquiring the geometric characteristics of the beams, even without correcting for the quenching effect. Indeed, depth–dose distributions acquired by the gels showed an excellent agreement with measured particle range with respect to reference values, with mean discrepancies of 0.5 ± 0.2 mm. Single-spot transverse FWHM values at increasing depths also presented an average agreement within 1 mm with values determined with radiochromic films, thus supporting the excellent spatial resolving capabilities of the dosimetric gel.

## 1. Introduction

In highly conformal radiotherapy, the need for 3D dosimetric measurement with high spatial resolution of the dose distributions has become even more important in the process of quality assurance (QA) and treatment plan verification [1,2]. Conventional dosimetric devices, such as ionization chambers or radiochromic films, are not able to directly measure three-dimensional dose distributions due to their 1D or 2D nature and often suffer from a poor tissue equivalence [3,4]. Hence, the interest in chemical gel dosimetry has surged. Throughout many studies, which have used conventional external beam radiotherapy techniques employing photons and electrons, promising results have been observed [2,5,6].

Hadron therapy (HT) is a kind of radiotherapy that makes use of accelerated hadrons (mainly protons and carbon ions) for the treatment of surgically inoperable, pediatric, radioresistant or recurrent tumors (retreatment) [7,8]. Due to the physical properties of these particles, a more conformal dose deposition is achieved compared to conventional radiotherapy, better sparing healthy tissues located around the tumor [9]. Up to now, few studies have been reported regarding the use of polymer gel dosimeters with high-LET particles. In these studies, good agreements have been observed in terms of geometrical dose information, while a strong suppression in dose–response in correspondence to the Bragg peak was measured due to a decrease in sensitivity attributable to quenching effects [10,11].

Polymer gel dosimeters are devices whose response is based on the radiation-induced polymerization of monomers infused in a gel matrix. Most notably, the degree of polymerization is locally proportional to the absorbed dose, and is three-dimensionally quantifiable by probing dose-induced alterations of physico-chemical properties. Examples of analytical techniques adopted to this scope include optical or X-ray computed tomography, ultrasoundography and, most commonly, magnetic resonance imaging (MRI) [12,13,14]. In particular, the use of an imaging technique allows for a direct measurement of the 3D dose distribution with a spatial resolution practically limited only by the performance of the measuring instrument [15]. Compared to traditional dosimetric systems, polymer gel dosimeters show an excellent radiological tissue equivalence and, unlike Fricke-type gel devices, the polymeric response is unaffected by the temporal and spatial diffusion of the dosimetric information, resulting in prolonged stability of the recorded signal [16,17,18].

This work focused on the characterization and study of the PAGAT gel for beam profile characterization in hadron therapy (HT) dosimetry. The PAGAT gel dosimeter, investigated for the first time by Venning et al. in 2004 [19], is a normoxic polyacrylamide and gelatin dosimeter (PAG) employing tetrakis hydroxymethyl phosphonium chloride (THPC) as an anti-oxidant [2]. Acrylamide (AAm) and N,N′-methylene-bis-acrylamide (BIS) are the monomers, mono-vinyl and divinyl respectively, whose polymerization is induced by the interaction with the radiation [20]. While AAm forms long, linear chains with no crosslinking, BIS is capable of forming several types of links (knots, loops, doublets) and is used as a crosslinking agent to increase the rigidity of the polymeric structure [21]. THPC acts as an anti-oxidant, scavenging O_2_ molecules which, due to their high radical affinity, would inhibit the polymerization mechanism [22]. Additionally to the standard PAGAT formulation, p-nitrophenol is incorporated as a polymerization inhibitor to reduce the sensitivity of the dosimeter, extend the linearity range and improve the long-term stability of the response [6,23]. The role of gelatin as a gelling agent is mainly to provide a three-dimensional matrix through which the polymers cannot diffuse, thus stabilizing the response of the dosimeter [24]. The dose information of an irradiated gel can be read by measuring changes of specific physical properties following the radiation-induced polymerization. MRI measurements and UV-Visible (UV-Vis) spectrophotometric analysis, which were employed during this study, are common readout methods [25,26]. Further details about these two techniques are presented in the Materials and Methods section.

The aim of the work was to investigate the new PAGAT formulation doped with p-nitrophenol following proton and carbon ion beam irradiation at variable particle extraction energies, to assess whether it could be suitable for volumetric beam profile characterizations in HT. Obtained geometric information, i.e., depth–dose curves and transverse profiles at variable depth, were then validated through comparison with data acquired with reference detectors.

Up to now very few studies have focused on the geometrical dosimetry abilities of polymeric gels in HT irradiations, as most of the existing literature investigates depth–dose profile measurements and the issue of quenching. As a key novelty aspect, this work focused specifically on investigating whether gel dosimetry could be applicable for geometric beam characterization without dedicated correction of the quenching effect.

## 2. Results and Discussion

### 2.1. Gel Dosimeter Response Characterization

**Figure 1:**
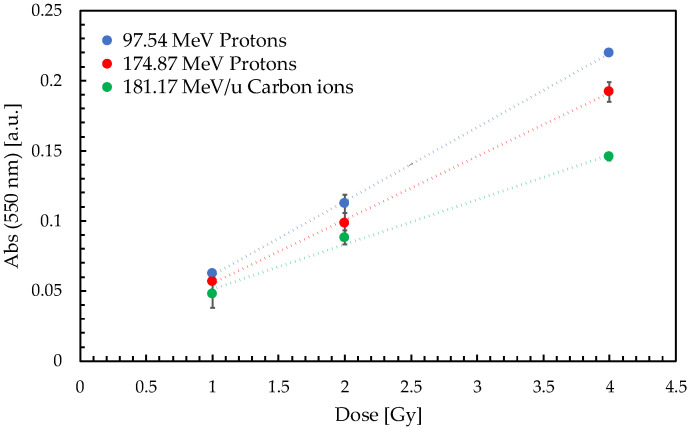
Net optical responses determined from characterization tests. Data were acquired 3 days after irradiation. Linear fitting curves are shown as dotted lines.

The purpose of this preliminary phase was to characterize the response of the dosimetric gels under different irradiation conditions in terms of linearity and temporal stability. The net optical response of the cuvettes irradiated with protons and carbon ions was recorded through spectrophotometric measurements and is reported in Figure 1.

A dose–response linearity (average R^2^ = 0.997 ± 0.004) was confirmed for all considered doses and beam types, with a mean dose accuracy of 3%. Mean intra-batch response uncertainties, evaluated across 4 replicates, were of 4%. MRI measurements also confirmed a linear response (average R^2^ = 0.995 ± 0.004) up to 4 Gy with a mean accuracy of 5%.

**Figure 2:**
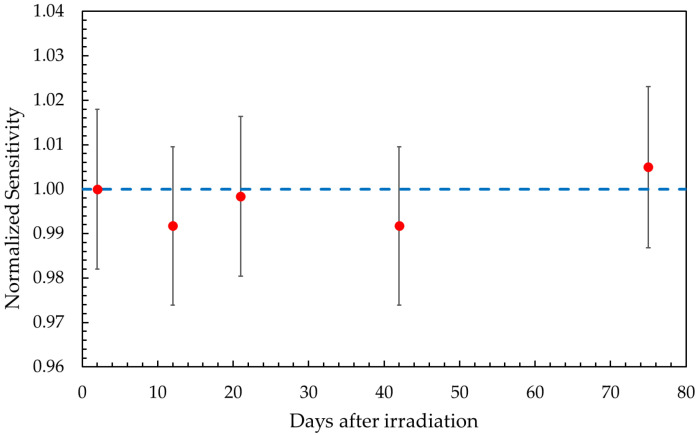
Temporal evolution of sensitivity for samples irradiated with 97.54 MeV protons and analyzed by UV-Vis spectrophotometry (red dots). Values are normalized with respect to the first measurement (dashed line).

The temporal stability of the gel response was evaluated by considering the evolution of the sensitivity over a period of up to 75 days post irradiation. After an initial period of response stabilization lasting approximately 5–10 h, all sensitivity values remained constant, regardless of irradiation condition, with maximum variations within ±1.5% over 75 days. Figure 2 illustrates the evolution of the sensitivity over this time period for samples irradiated with 97.54 MeV protons. Trends for other irradiation conditions were essentially identical. A long-term stability of the chemical response can thus be inferred, supporting the robustness of the dosimetric system against analysis delays.

### 2.2. Volumetric Response

**Figure 3:**
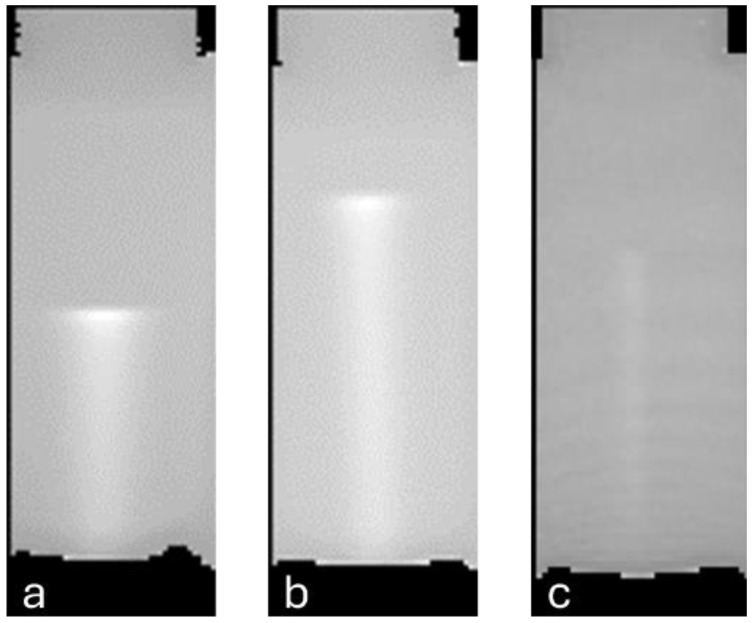
Greyscale R_2_ maps for cylindrical phantoms sliced along a plane intersecting beam axis. R_2_ maps correspond to the 97.54 MeV proton beam (**a**), 118.20 MeV proton beam (**b**) and 208.58 MeV/u carbon ion beam (**c**). It is possible to appreciate the difference in lateral diffusion between the carbon ion beam and proton beam. Each pixel has a size of 1.4 × 1.4 mm^2^.

The analysis of cylindrical phantoms aimed at assessing the volumetric response of PAGAT gels. Dose profiles along the beam axis and transversal dose distributions at increasing depths were assessed and benchmarked against reference curves in terms of particle range and full-width half-maximum (FWHM). Three phantoms were irradiated with single-spot beams: two with protons at 97.54 MeV and 118.20 MeV, and one with carbon ions at 208.58 MeV/u. Non-irradiated gel phantoms were also imaged to provide a blank value for subtraction. The standard deviation of the MR signal in a central portion of a blank sample was assessed at 0.9%, indicating good uniformity of the gel. Reconstructed longitudinal slices of the R_2_ maps for the three phantoms are illustrated in Figure 3.

#### 2.2.1. Depth–Dose Profiles

The depth–dose profiles were extracted from the R_2_ maps by selecting average voxel signals in the central axial region of the phantoms on the longitudinal slice intercepted by the beam axis. The bottom of the phantoms (inlet of the beam) was chosen as the origin of the depth axis, while the mean R_2_ value of a non-irradiated portion of the gel was taken as origin of the R_2_ axis and subtracted as a blank. Dose profiles measured by the gel phantoms were compared with reference profiles measured using ionization chambers in water, whose maxima were set to one for easier quantification of the quenching effect. Due to signal quenching, the maxima of the profiles measured by gel phantoms could not be normalized at one and therefore a matching between gel and reference profiles was imposed at an appropriate depth to make the profile plateau of the two curves correspond. This methodology has already been reported in the literature [10,11,27], and is useful in highlighting the response of the gel in the high-LET portion of the beam by imposing response normalization in the low-LET plateau where quenching effects are negligible.

**Figure 4:**
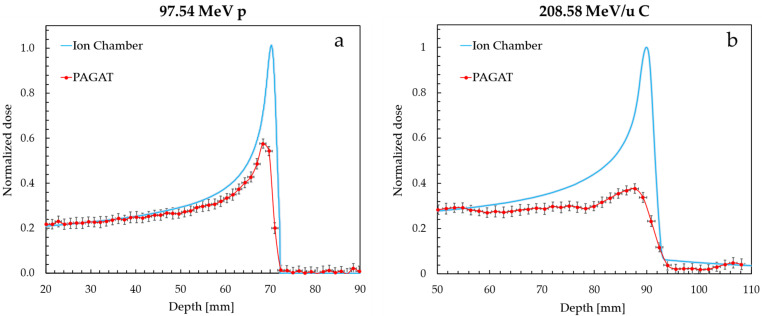
Normalized depth–dose profile for 97.54 MeV (70 mm range in water) proton single-spot beam (**a**) and 208.58 MeV/u (90 mm range in water) carbon ion single-spot beam (**b**). The two curves were normalized at a depth of 20 mm and 50 mm, respectively. Uncertainty for ion chamber data is represented by the shaded area.

In the case of 97.54 MeV proton irradiation (Figure 4a), the agreement between the gel and reference curves is, within their respective uncertainties, good at the plateau up to a depth of 40 mm. At the Bragg peak, however, the gel underestimates the absorbed dose of 40% compared to the reference profile. This under-response confirms the presence of a quenching effect due to LET increase in the distal part of the beam as expected from the literature results [10,28].

The presence of quenching effects also hinders a comparison of measured peak depths as these are defined based on the maximum or nearby values of the relative dose curve. However, by defining an effective peak position as the depth of the 2% signal (R_2%_), an agreement within 0.2 mm is found between the gel phantom and reference data. This result is even more notable when compared to the spatial resolution of MRI, i.e., 1.4 × 1.4 × 1.4 mm^3^.

Similar results were obtained for the 118.20 MeV proton irradiation, which resulted in a measured depth–dose profile which agrees with the reference one in the plateau region up to a 60 mm depth. In this instance the underestimation at the Bragg peak is 55%, greater than the previous case. The LET dependence of the dosimeter sensitivity appears to be affected by the energy of the particles. Similarly to the 97.54 MeV proton irradiation, also in this second case the particle range recorded by the gel shows a small 0.6 mm difference in terms of R_2%_ values with respect to the reference data.

The 208.58 MeV/u carbon ion irradiation profile (Figure 4b) shows similar characteristics to the previous ones. From 55 mm onwards, the quenching effect becomes relevant as the LET increases at higher depths. The underestimation at the Bragg peak is 60%, the largest of all three cases. Due to the presence of a dose tail in the distal part of the ion depth–dose curve, which results from the projectile fragmentation, an effective peak position of the signal at the depth of 6% (R_6%_) was defined for the carbon ion irradiation. The dose tail is visible in the gel profile in spite of an initial slight underestimation, and the depth position of the peak and its tail are nonetheless well recorded by the gel dosimeter.

In Table 1 the numerical R_2%_ and R_6%_ values measured by gel phantoms are compared with the reference ones. An average discrepancy of 0.5 ± 0.2 mm was recorded across all irradiation conditions, which is comparable to the spatial resolution of the dosimetric system.

#### 2.2.2. Transverse Dose Distributions

**Figure 5:**
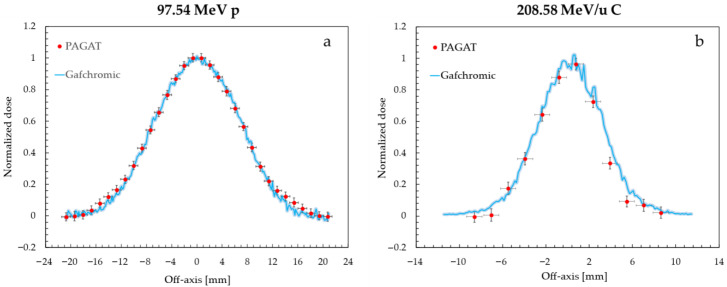
Transverse dose distribution for 97.54 MeV proton (**a**) and 208.58 MeV/u carbon ion single-spot beams (**b**). The curves were normalized to their values at 0 mm off-axis. The gel profiles were sampled at a 20 mm depth from the bottom of the phantom. The shaded area for the Gafchromic profiles represent ± 1σ uncertainty.

The R_2_ maps obtained from MRI were sampled along directions perpendicular to the beam axis at various depths and the resulting transverse dose distributions were compared to experimental profiles recorded by radiochromic films. The transverse profiles measured for the 97.54 MeV proton and 208.58 MeV/u carbon ion beam are shown in Figure 5, while Table 2 presents the FWHM measured by gel phantoms at different depths compared to the reference ones. Where necessary, linear interpolation between data points crossing the 50% relative dose value was adopted in order to determine the FWHM. Uncertainties for FWHM values measured by PAGAT gel and Gafchromic films were dependent mainly on the spatial resolution of the devices, and amounted to ± 0.7 mm and ± 0.2 mm respectively.

Reported sampling positions were selected to analyze increasing depths of the dose distribution. Regardless of sampling depth, profiles acquired by gels and films presented good agreement, as confirmed by an average discrepancy between FWHM of <1 mm, and maximum differences of <1.5 mm. Even in the case of the C ion profiles, where the finite spatial resolution of the MRI analysis is of the same order of magnitude of the profile FWHM, agreement with reference values is still remarkable at <0.2 mm.

## 3. Conclusions

This study analyzed a new PAGAT formulation that incorporates p-nitrophenol as a polymerization inhibitor, which was previously investigated only using photon and electron irradiation. For the first time, the new formulation has been studied in a Hadron Therapy facility. The present study demonstrated the suitability of the dosimetric gel in performing geometric beam characterizations for machine QA purposes without the need for correcting quenching effects, also in the case of carbon ion beams. Achieved levels of accuracy and spatial resolution are compliant with machine QA requirements [29], and could be further refined by improved MRI scanning sequences.

Also, a stability analysis of PAGAT dosimeters demonstrated similar results to those irradiated with photons or electrons [22,30,31], suggesting that the particle type does not significantly affect this parameter. The measured particle ranges and FWHM were in agreement with those acquired with reference devices. The temporal stability due to the absence of diffusion-related signal degradation makes the PAGAT dosimeter a reliable recording medium for months after irradiation. The lack of need for quenching correction also greatly simplifies the applicability of the dosimetric gel system in routine QA protocols.

Despite the positive results, the main drawbacks of this device are still represented by the quenching effect in high-LET regions, which negates the possibility of performing patient-specific QA, as well as the time required by the gel to fully develop its chemical response, which retards analysis by several hours from irradiation. Future developments in gel dosimetry for HT should therefore address the issue of high-LET quenching. Possible strategies include constructing LET calibration curves [32] to correct the under-response at the Bragg peak or exploring new gel compositions with reduced LET dependence [33], also considering heavy particles. Standardized and optimized MRI sequences should also be defined to fully exploit the 3D spatial resolution of gel dosimeters by reducing imaging artifacts and improving the signal-to-noise ratio.

## 4. Materials and Methods

The PAGAT gel composition and its preparation protocol were obtained from the literature [19]. As suggested by previous studies, p-nitrophenol was incorporated as a polymerization inhibitor to reduce the sensitivity of the dosimeter, extend the linearity range and improve the long-term stability of the response [6,23]. To improve the scavenging ability of THPC, it is important that deionized water is employed as a solvent during preparation in order to avoid reactions of THPC with contaminants [22]. Table 3 summarizes the composition of the gels prepared in this work. All reagents were purchased from Merck KGaA (Darmstadt, Germany).

### 4.1. Gel Dosimeter Preparation

For the preparation of the dosimetric gel, the following procedure was adopted [6]:

- Dissolve AAm and BIS in ≈55% of the overall deionized water volume while heating at 50 °C and stirring;
- Meanwhile, add porcine skin gelatin to the remaining 45% of water volume while heating at 50 °C and stirring;
- Allow both solutions to cool down to 30 °C while stirring;
- Once cooled, slowly add the AAm/BIS solution to the gelatin one under constant stirring;
- Add p-nitrophenol and THPC dropwise under stirring.

**Figure 6:**
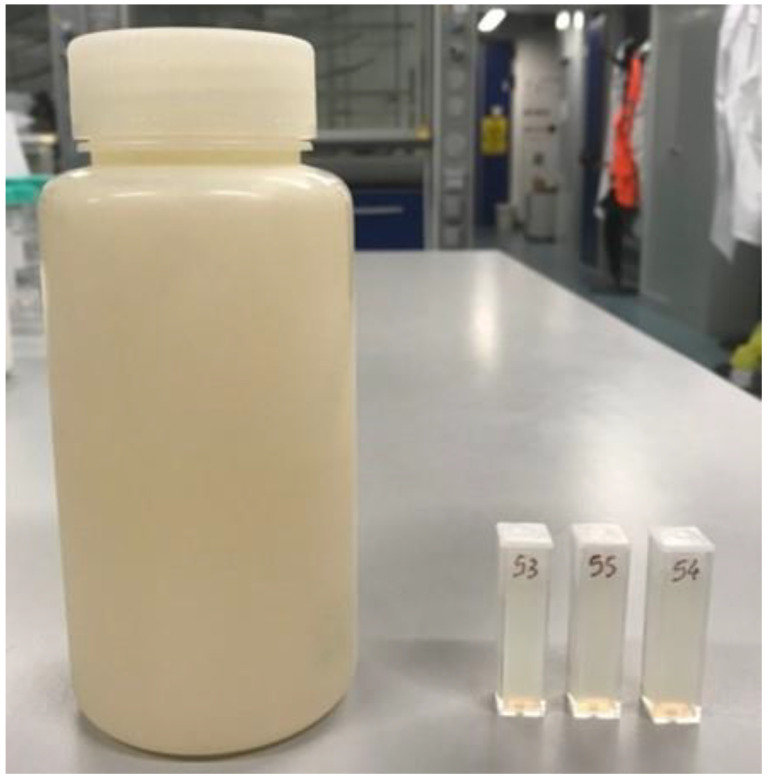
Cylindrical gel phantom next to three spectrophotometric cuvettes used for characterization. External cuvette dimensions: 11 × 11 × 50 mm^3^. External phantom dimensions: 75 mm diameter, 165 mm height.

The resulting dosimetric solution was finally poured into different containers based on their intended use, as illustrated in Figure 6. In particular, samples for calibration and stability assessment consisted of 5 mL PMMA spectrophotometric cuvettes. Phantoms for single-spot volumetric analyses were prepared in 0.5 L HDPE bottles with screw tops. After preparation, all samples were stored in a refrigerator at 7 °C to allow the gelatin to solidify. Irradiation was performed at least 12 h after preparation to ensure the stabilization of the dosimetric composition [34].

### 4.2. Irradiation

The gel dosimeters were irradiated at the Centro Nazionale di Adroterapia Oncologica (CNAO) facility in Pavia, Italy, with monoenergetic proton and carbon ion beams generated by the synchrotron accelerator, whose extraction energy ranges from 62.73 to 228.57 MeV for protons beams and from 115.23 to 398.84 MeV/u for carbon ions [7,29], using the active spot scanning beam delivery technique, with beam intensity (i.e., dose rate) fixed at approximately 3.0 × 10^9^ and 6.0 × 10^7^ particles per second for protons and carbon ions, respectively.

**Figure 7:**
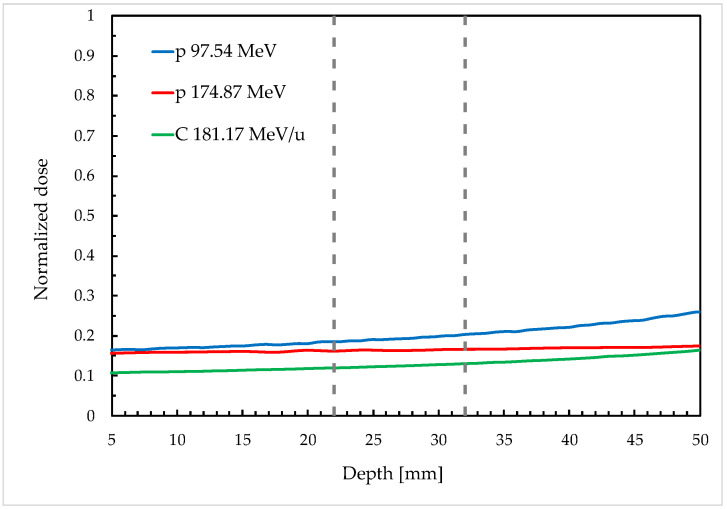
Illustration of longitudinal dose profiles of the three irradiation conditions employed with cuvette samples. The sensitive volume occupied by the gel is represented by the area between the two dashed lines.

**Figure 8:**
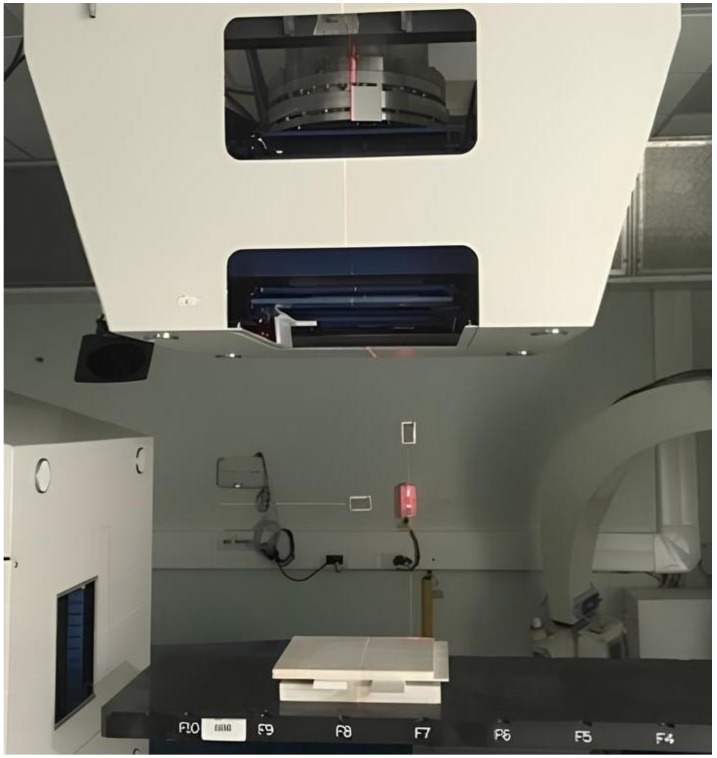
Irradiation setup for the cuvettes using a vertical beamline. The 30 × 30 cm^2^ RW3 slabs surrounding the samples are positioned on top of the treatment couch.

All gel samples were allowed to reach room temperature (22 ± 1 °C) before irradiation. To assess the repeatability of response, the cuvettes were positioned in groups of four, with their major axis orthogonal to the beam direction, in a RW3 (PTW Freiburg) slab phantom. A dedicated insert was used to position the samples with the center of their sensitive volume at 2.7 cm of water-equivalent depth in the phantom, i.e., at the depth–dose profile plateau (see Figure 7), to minimize dose gradients in the longitudinal direction and allow the groups of four samples to be irradiated uniformly. The irradiation configuration is shown in Figure 8. The response characterization phase considered doses of 1 Gy, 2 Gy and 4 Gy using monoenergetic protons with extraction energies of 97.54 MeV (corresponding to a Bragg peak at a 70 mm depth in water) and 174.87 MeV (201 mm range in water), as well as monoenergetic carbon ions of 181.17 MeV/u (90 mm range in water). For the adopted irradiation setup, the longitudinal dose uniformity in the gel was 2.6%, 0.9% and 2.5% respectively for the selected beams.

The cylindrical phantoms were irradiated along their longitudinal axis using single-spot beams. Three phantoms were irradiated: two using monoenergetic protons at energies 97.54 MeV and 118.20 MeV, and one using a monoenergetic carbon ion beam of 208.58 MeV/u energy. Particle ranges in water were respectively 70 mm, 101 mm and 90 mm. Doses to the peak ranged from 2.1 Gy to 3.1 Gy.

Before the irradiation of the samples, accelerator outputs were characterized using a Farmer ionization chamber [35]. All samples were located in their nominal irradiation positions thanks to a three-axis laser alignment system, guaranteeing a positioning reproducibility and accuracy of <1 mm.

### 4.3. Dosimeter Analysis

UV-Vis spectrophotometry and MRI were used for cuvette analysis, while MRI exclusively was used for cylindrical phantom investigation. All measurements were performed at least two days after irradiation to allow the polymerization response to develop and stabilize [31].

MRI scanners are used to map changes in the longitudinal and transversal relaxation rates (R_1_ and R_2_) of the magnetization vector through a sequence of radio frequency (RF) pulses. As the relaxation rates are proportional to the absorbed dose, a correlation between the two quantities can be established through a proper calibration [36]. The temporal evolution of the relaxation mechanism shows an exponential behavior for magnetization vector components associated with two relaxation times. The dose–response in terms of R_2_ for gelatin-based polymer dosimeters is more pronounced with respect to R_1_ and, consequently, T_2_-weighted images are usually acquired for the scanning of these devices [36]. To measure the R_2_ relaxation rate of the polymer gel dosimeter, a multiple-spin echo sequence is commonly employed. It consists of a series of RF pulses inducing a train of echo signals generated after an echo time (TE); R_2_ values in each voxel are then obtained by fitting an exponential decay curve of the corresponding voxel intensities versus the sequence echo times [37]. Subsequently, through a process of calibration, the absorbed dose can be correlated to the relaxation rate measured in each voxel, thus obtaining a dose image of the irradiated dosimeter. In contrast, UV-Vis spectrophotometry is a technique that aims at measuring the optical absorption property of the sample to quantify the changes of the irradiated chemical dosimeter [38]. The absorption measurement is performed by illuminating the sample with monochromatic light and measuring the transmitted intensity which is then compared with the intensity of light passing through a reference sample. For an irradiated polymeric gel system, the optical absorbance is proportional to the absorbed dose [23].

Optical analysis of the cuvettes utilized a LAMBDA 650 UV/Vis spectrophotometer (Perkin Elmer, Springfield, IL, USA), at a sampling wavelength of 550 nm and an integration time of 0.2 s. The goal of this analysis was to construct dose-absorbance curves, characterizing the polymerization response in terms of linearity, dose accuracy and temporal stability. For this purpose, the mean absorbance of blank non-irradiated specimens was subtracted from that of irradiated samples, and a linear interpolation of dose values was obtained. The temporal stability of the gel response was assessed by repeated measurements over a two-month period post-irradiation, during which samples were stored under refrigeration.

Magnetic resonance analyses were conducted using a 3 T Magnetom Skyra Fit MRI scanner (Siemens, Munich, Germany). Samples were allowed to thermalize up to room temperature (22 ± 1 °C) before proceeding with the analysis. Cylindrical phantoms were scanned using MRI analysis in order to capture the three-dimensional dose deposition information of the single-spot irradiations. A multi-slice multi-echo pulse sequence [39] was used for all dosimeters involving 32 echo times (TE) ranging from 20 to 640 ms, with 20 ms increments. The reconstructed voxel dimensions were 1.4 × 1.4 × 1.4 mm^3^. The MRI raw data comprised T_2_-weighted images capturing signal intensity at different echo times and an image reconstruction algorithm in the Matlab R2024a (The MathWorks, Inc., Natick, MA, USA) environment was adapted to generate R_2_ maps. The algorithm involved several steps aimed at fitting the exponentially decaying signal intensity pixel-by-pixel using maximum-likelihood estimation with χ^2^ minimization and constructing R_2_ maps of the scanned objects [40].

### 4.4. Data Analysis

For the analysis of the cuvettes, the coefficients of determination R_2_ were calculated following a linear interpolation of the experimental data to evaluate the linearity of the optical and MRI response. Accuracy, given by the relative difference between the measured dose compared to the prescribed one, was also computed.

The analysis performed on the cylindrical phantoms was intended to evaluate the three-dimensional performance of the gel by comparing measured depth–dose curves and transverse distributions of the single-spot irradiations with reference profiles obtained with an ionization chamber (PeakFinder, PTW, Zurich, Switzerland) and EBT3 gafchromic films, properly calibrated for proton and carbon ion beam irradiation [41]. Values of the particle range and FWHM were determined through linear interpolation between adjacent data points. Uncertainties associated with such values were calculated by also taking into account the finite spatial resolution of MRI and Gafchromic images.

## Figures and Tables

**Table 1:** Numerical comparison of R_2%_ values for proton irradiations and R_6%_ for carbon ion irradiation. Last column shows the difference between the PAGAT and reference values.

	PAGAT	Ion Chamber	Difference
97.54 MeV p	72.4 ± 0.7 mm	72.2 ± 0.2 mm	0.2 mm
118.20 MeV p	104.5 ± 0.7 mm	103.9 ± 0.2 mm	0.6 mm
208.58 MeV/u C	94.0 ± 0.7 mm	93.4 ± 0.2 mm	0.6 mm

**Table 2:** Numerical comparison of the FWHM values obtained by the two dosimetric systems for different proton and carbon ion irradiations measured at different depths d. Uncertainties for FWHM values measured by PAGAT gel and Gafchromic films were ± 0.7 mm and ± 0.2 mm respectively.

		FWHM		
	Depth	PAGAT	Gafchromic	Difference
97.54 MeV p	d = 20 mm	15.9 mm	15.8 mm	0.1 mm
	d = 35 mm	16.1 mm	16.9 mm	−0.8 mm
	d = 66 mm	17.5 mm	17.8 mm	−0.3 mm
118.20 MeV p	d = 20 mm	13.2 mm	13.7 mm	−0.5 mm
	d = 50 mm	14.0 mm	15.3 mm	−1.3 mm
	d = 96.5 mm	16.1 mm	17.5 mm	−1.4 mm
208.58 MeV/u C	d = 20 mm	6.4 mm	6.6 mm	−0.2 mm
	d = 45 mm	6.7 mm	6.8 mm	−0.1 mm
	d = 80 mm	7.5 mm	7.7 mm	−0.2 mm

**Table 3:** PAGAT gel composition. Ppm (parts per million) refers to mass fraction.

Compound	Concentration
AAm	3% wt.
BIS	3% wt.
Gelatin	5% wt.
Deionized water	89% wt.
p-nitrophenol	2.5 ppm
THPC	10 mM

## Data Availability

The original contributions presented in this study are included in the article. Further inquiries can be directed to the corresponding author.
